# Internally Guided Lower Limb Movement Recruits Compensatory Cerebellar Activity in People With Parkinson's Disease

**DOI:** 10.3389/fneur.2019.00537

**Published:** 2019-06-07

**Authors:** Jonathan H. Drucker, K. Sathian, Bruce Crosson, Venkatagiri Krishnamurthy, Keith M. McGregor, Ariyana Bozzorg, Kaundinya Gopinath, Lisa C. Krishnamurthy, Steven L. Wolf, Ariel R. Hart, Marian Evatt, Daniel M. Corcos, Madeleine E. Hackney

**Affiliations:** ^1^Atlanta VA Center for Visual and Neurocognitive Rehabilitation, Decatur, GA, United States; ^2^Department of Neurology, School of Medicine, Emory University, Atlanta, GA, United States; ^3^Departments of Neurology, Neural and Behavioral Sciences, and Psychology, Pennsylvania State University, Hershey, PA, United States; ^4^Department of Psychology, Georgia State University, Atlanta, GA, United States; ^5^Health and Rehabilitation Science, University of Queensland, Brisbane, QLD, Australia; ^6^Department of Radiology and Imaging Sciences, Emory University, Atlanta, GA, United States; ^7^Department of Physics and Astronomy, Georgia State University, Atlanta, GA, United States; ^8^Division of Physical Therapy, Department of Rehabilitation Medicine, School of Medicine, Emory University, Atlanta, GA, United States; ^9^Department of Cell Biology, School of Medicine, Emory University, Atlanta, GA, United States; ^10^Division of General Medicine and Geriatrics, Department of Medicine, School of Medicine, Emory University, Atlanta, GA, United States; ^11^Physical Therapy and Human Movement Sciences, Northwestern University, Chicago, IL, United States

**Keywords:** Parkinson's, putamen, cerebellum, lower limb, internally guided, externally guided, striatum, fMRI

## Abstract

**Background:** Externally guided (EG) and internally guided (IG) movements are postulated to recruit two parallel neural circuits, in which motor cortical neurons interact with either the cerebellum or striatum via distinct thalamic nuclei. Research suggests EG movements rely more heavily on the cerebello-thalamo-cortical circuit, whereas IG movements rely more on the striato-pallido-thalamo-cortical circuit (1). Because Parkinson's (PD) involves striatal dysfunction, individuals with PD have difficulty generating IG movements (2).

**Objectives:** Determine whether individuals with PD would employ a compensatory mechanism favoring the cerebellum over the striatum during IG lower limb movements.

**Methods:** 22 older adults with mild-moderate PD, who had abstained at least 12 h from anti-PD medications, and 19 age-matched controls performed EG and IG rhythmic foot-tapping during functional magnetic resonance imaging. Participants with PD tapped with their right (more affected) foot. External guidance was paced by a researcher tapping participants' ipsilateral 3rd metacarpal in a pattern with 0.5 to 1 s intervals, while internal guidance was based on pre-scan training in the same pattern. BOLD activation was compared between tasks (EG vs. IG) and groups (PD vs. control).

**Results:** Both groups recruited the putamen and cerebellar regions. The PD group demonstrated less activation in the striatum and motor cortex than controls. A task (EG vs. IG) by group (PD vs. control) interaction was observed in the cerebellum with increased activation for the IG condition in the PD group.

**Conclusions:** These findings support the hypothesized compensatory shift in which the dysfunctional striatum is assisted by the less affected cerebellum to accomplish IG lower limb movement in individuals with mild-moderate PD. These findings are of relevance for temporal gait dysfunction and freezing of gait problems frequently noted in many people with PD and may have implications for future therapeutic application.

## Introduction

Parkinson's disease (PD) is a neurodegenerative disorder leading to motor symptoms including impaired lower limb control, bradykinesia, freezing, and postural instability, as well as cognitive impairment and other non-motor symptoms. Mobility programs [e.g., mobility training, partnered dance (e.g., tango) and non-partnered dance (e.g., Dance for PD), tandem biking, tai chi] are effective for improving motor function in people with PD (3–7). These programs use a mix of externally guided (EG) and internally guided (IG) (1) movement strategies, both of which have evidence supporting their use in rehabilitative scenarios. However, little is known about whether EG or IG approaches are more effective in PD, and the relevant neural processes are not clearly understood.

In healthy people, functional magnetic resonance imaging (fMRI) studies have suggested distinct neural pathways for upper limb IG vs. EG movements in a variety of contexts (1, 8–14). PD affects both IG and EG movements. However, due to dysfunction of the striato-pallido-thalamo-cortical (STC) circuit, people with PD have particular difficulty with IG tasks (2, 15, 16). Because EG movements likely rely on relatively spared brain regions, at least in early stages of PD, the patterns of neural activation during EG tasks differ little between those with PD and controls, whereas there are distinct differences between groups during IG task performance (17). Proper completion of IG movements relies on efficient function of subcortical loops involving the basal ganglia (18, 19). The most immediate impact of dysfunction in IG movement in PD relates to impaired cortico-striatal communication during movement initiation. One behavioral manifestation of this disruption is the presence of akinesia in PD during IG movement (20). Cortical initiation of IG movements relies on modulation and signal augmentation that is highly sensitive to disruption when filtered through dysfunction of the basal ganglia (21). Under IG conditions, the cerebello-thalamo-cortical (CTC) pathway may be recruited more in people with PD than in neurotypical subjects, possibly denoting recruitment of EG circuitry, which is more robust to deterioration in PD than dopamine-dependent STC pathways (22). In keeping with the idea of increased compensatory activity/connectivity of cerebellar circuits during IG tasks, STC connections are weaker in individuals with PD than in controls, whereas CTC connections are stronger (2).

Visual, auditory, tactile, and attentional cues have all been used experimentally to examine the effects of external guidance in PD. Overwhelming evidence demonstrates benefits of visual and auditory cueing in behavioral studies for people with PD (23), and such cues are often incorporated in physical therapy (24). Tactile cues have been studied less; yet they may be processed faster and more efficiently, with less attentional demand, than visual and auditory cues (25–28). Somatosensory integration for prioritizing tactile and proprioceptive feedback is not disrupted by PD (26). Somatosensory cueing can supersede visual distractors (28). Using haptic speed cues from a moving handrail, people with PD increased stride length (25). Tactile cues decreased timing errors during a dual task (27). Rhythmic somatosensory cues increase turning speed and may be more effective than visual cues (29). Interestingly, humans can abstract a pattern of beats from tactile rhythms as efficiently as from auditory patterns (30).

Using fMRI, differences have been observed in the neural control of upper and lower extremity movement, including greater lateralization for upper compared to lower limb movements (14, 31, 32). However, studies specifically investigating neural correlates of movements of the lower extremity, particularly in the context of IG vs. EG control, are less common, especially in populations with neurological pathology like PD. Schwingenschuh and co-workers, using a simple EG ankle dorsiflexion task, found that both people with PD and healthy controls activated lobules I-V in the ipsilateral cerebellum during EG movement (33). Spraker et al. further noted increased recruitment of cerebellar structures and pathways with disease progression in PD (34, 35).

Knowledge about the effect of PD on neural circuits with respect to rhythmic EG or IG movements of the *lower extremity* is incomplete. The purpose of this study was to investigate neural correlates of IG and EG movements using rhythmic foot tapping in people with PD vs. age-matched neurotypical (NT) controls. We developed a task that is related to the foot-tapping test used in clinical practice from the Unified Parkinson's Disease Rating Scale (UPDRS), and which we adapted for fMRI to specifically assess lower limb rhythmic motor control. We used a marginally more complex rhythmic task than previous studies for use in future rehabilitative scenarios. We hypothesized that: (1) like the upper limb literature, the striatum would be less active in PD than controls, and (2) the cerebellum would be adaptively recruited for IG tasks by people with PD.

## Materials and Methods

The institutional review board at Emory University School of Medicine and the Research and Development Committee of the Atlanta VA Health Care System approved this work. Participants provided written informed consent before participating.

### Participants and Initial Assessments

Forty-one individuals (18 men, 23 women) were recruited. Inclusion criteria required participants be ≥40 years old and able to walk ≥3 meters with or without assistance. PD participants were recruited through the VA Informatics and Computing Infrastructure database, as well as support groups, educational meetings, newsletters, physician referrals, word of mouth, outreach events and research websites. Nineteen NT participants, age-matched to PD patients (6 men, 13 women; *M* age 64.7, *SD* = 10.2), had no history of neurological or psychiatric disorders. Twenty-two PD participants (12 men, 10 women; *M* age 67.7, *SD* = 9.9) were recruited. All participants were right handed as verified by the Edinburgh handedness survey. No participants had contraindications to undergo MRI. All PD participants were clinically diagnosed with PD by a movement disorders specialist based on the United Kingdom PD Society Brain Bank diagnostic criteria (36, 37). These patients had unilateral onset of symptoms, displayed clear symptomatic benefit from antiparkinsonian medications, e.g., levodopa (38), and were in Hoehn and Yahr stages I-III. Patients who had a tremor score >1 in either lower limb and/or moderate-severe head tremor were excluded. PD participants were tested in the OFF state, i.e., ≥12 h after their last dose of anti-parkinsonian medication. Participants' Levodopa Equivalent Daily Dosage was calculated with standardized procedures (39). Twenty-two participants were on carbidopa/levodopa including two individuals who were additionally taking an extended release version of this medication at night before bed. The most common formulation was ER 25/100. Three participants were taking Amantadine, and three people were taking Azilect. Two people were taking Mirapex, Ropinirole HCL, or Trihexyphenidyl. One person was taking Comtan, and one person was taking Entacapone. Twelve participants reported taking more than one PD medication.

Participants were evaluated for general health and ability to perform activities of daily living (ADLs) with the Composite Physical Function index (CPF) (40) Participants scored ≥19 on the Montreal Cognitive Assessment (41), excluding individuals with dementia (42). The Beck Depression Inventory-II (BDI-II) assessed depression (43). A score of ≥30, indicating severe depression, was a cutoff for the BDI-II. Focusing on lower-limb function, participants were administered mobility measures including Dynamic Gait Index (DGI) (44), the Single Timed Up and Go Test (TUG) (45) with cognitive (TUG-c, counting backwards by 3 s) and manual (TUG-m, carrying a full glass of water) conditions, and preferred and fast gait (46). PD participants also completed the Freezing of Gait questionnaire and were administered the Fullerton Advanced Balance Scale (47) and the Movement Disorders Society UPDRS (MDS-UPDRS) (48) parts I-IV to derive a total score and a subscale III score. PD and NT group characteristics are found in Table 1.

**Table 1:** Characteristics of participants.

	**NT [*****n*** **=** **19; (13 F)]**				**PD [*****n*** **=** **22, (10 F)]**				**Between-group statistics**	
**Variable**	**Mean**	***SD***	**Min**	**Max**	**Mean**	***SD***	**Min**	**Max**	***t***	***p***
Age (year)	64.7	10.2	43.0	82.0	67.7	9.9	41.0	85.0	0.95	0.348
Montreal Cognitive Assessment (/30)	26.9	2.8	19.0	30.0	26.5	3.2	20.0	30.0	−0.39	0.701
Time with Diagnosed PD (year)	–	–	–	–	6.0	4.3	0.0	18.0	–	–
UPDRS-Total Score	–	–	–	–	64.5	19.7	41.0	99.0	–	–
UPDRS-III score	–	–	–	–	33.9	9.5	21.0	55.0	–	–
Hoehn & Yahr Staging	–	–	–	–	2.3	0.6	1.0	3.0	–	–
Freezing of Gait questionnaire					6.5	4.7	0	15		
Levodopa Equivalent Daily Dosage (mg)					663	346	300	2150		
Beck Depression Inventory-II	8.3	12.0	0.0	54.0	10.3	5.8	2.0	25.0	0.65	0.522
Trails B-A (seconds)	57.5	41.4	11.0	160.7	53.8	51.4	4.1	199.9	−0.26	0.799
Fullerton Advanced Balance scale	–	–	–	–	26.2	10.3	0.0	38.0	–	–
Timed Up & Go single (s)^*^	7.4	1.9	5.0	11.6	9.6	3.3	5.5	18.9	2.57	0.015
Timed Up & Go cognitive (s)^*^	10.8	4.0	5.4	20.8	13.9	5.1	7.5	22.3	2.13	0.040
Timed Up & Go dual-task errors	0.3	0.6	0.0	2.0	0.5	0.7	0.0	2.0	0.80	0.428
Timed Up & Go dual-task correct^*^	4.0	2.0	1.0	8.0	6.1	2.7	1.0	11.0	2.69	0.011
Preferred Gait^*^ Speed (m/s)^*^	1.3	0.2	0.8	1.7	1.0	0.3	0.4	1.6	−3.63	0.001
Fast Gait Speed (m/s)^*^	1.8	0.5	1.0	2.6	1.4	0.4	0.5	2.0	−2.90	0.007
Dynamic Gait Index (/24)	20.4	5.5	0.0	24.0	18.4	5.3	2.0	23.0	−1.22	0.230
Number of Comorbidities	2.5	2.1	0.0	9.0	3.4	1.6	1.0	7.0	1.47	0.150
Composite Physical Function index^*^	22.8	2.9	12.0	24.0	18.8	5.8	6.0	24.0	−2.87	0.007

*Independent t tests were run between groups*.

* *p < 0.05. A chi square statistic was used to compare the sex distribution in the groups, with Chi-square statistic = 2.18, p = 0.14, indicating no sex differences between groups. These characteristics show participants were similar in age and global cognition, but PD participants had gait impairments and comparatively more trouble with ADLs*.

### Behavioral (Foot-Tapping) Task During Imaging

**Figure 1:**
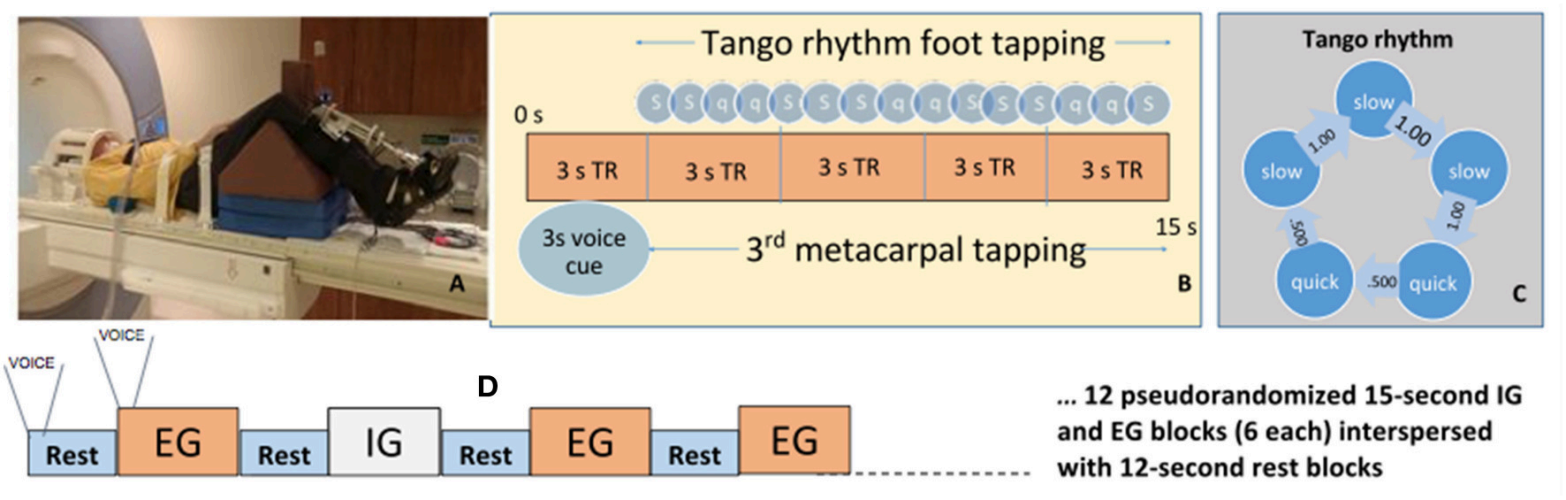
Activation paradigm and participant fMRI setup. **(A)** Participant prepped for scanner. **(B)** Ideal foot-tapping sequence within each active block. **(C)** The slow, slow, quick, quick, slow tango rhythm was used as the target rhythm for participants. **(D)** Activation paradigm for internally and externally guided blocks. Voice, voice cue.

Laying supine, participants performed a foot-tapping task with an orthopedic wedge under the knees to maintain a 90° angle and isolate ankle movement (Figure 1A). They wore a custom-made instrumented ankle orthotic on the leg *more* affected by PD based on leg agility and foot tapping items of the MDS-UPDRS-III. For equal bilateral rating, patient-reported side of parkinsonian symptom onset was factored. We included only PD participants most affected on their right side. Controls tapped with their right foot. Participants were told to dorsiflex, then plantarflex till they reached a stop. The arc between stops, largest possible range of motion (ROM) was 20 degrees. The orthotic affixed to the lower extremity incorporated position measurement, and sampled foot position continuously (2,000 Hz). Custom software (BioMaq) built with LabView (National Instruments, Austin, TX) acquired data.

Before scanning, participants learned a 4 s rhythmic sequence designed to approximate a basic rhythm of the Argentine tango dance: slow, slow, quick, quick, slow (Figure 1B). The tango rhythm was chosen to present a more complex rhythm than previous studies and for its current and future use in rehabilitation, e.g., with adapted tango therapies (49). Intervals between taps were 1 s, 1 s, 0.5 s, 0.5 s, 1 s (Figure 1C). All participants were trained ~20 min on the task while lying on the scanner table. Examiners verified that the participant fully understood the task and could perform the EG, IG, and Rest conditions proficiently and as instructed before beginning the scanning session. **EG condition blocks**: participants tapped their foot immediately after detecting a finger tap on their 3rd metacarpal head from an examiner listening to an audible version of the rhythm via headset. The examiner was placed just outside the scanner bore next to the patient table on the right side of the participant. The examiner ensured that they could comfortably reach the hand of the participant prior to the scan. This individual was extensively trained on delivering the taps to the participant such that the delivered taps matched the auditory timing provided with the headset. **IG condition blocks:** participants tapped the rhythm as practiced prior to the scan without prompting tactile cues on their hand. The examiner instead tapped the 3rd metacarpal head *after* each participant foot-tap to control for the experience of tactile sensation in the EG and Rest conditions. Participants were told to not pay attention to the taps on their hand during the IG condition. **Rest condition:** Examiners tapped the participants' 3rd metacarpal head at 1 Hz in order to control for the experience of the tactile sensation during the IG and EG conditions. There were twenty-four 15 s blocks of EG, and twenty-four 15s blocks of IG tapping across four runs. Block order was randomized in each functional run. Throughout the first 3 s of each block, a voice cue indicated whether the participant would experience an EG or IG trial. With 12 s rest blocks interposed between task, tapping lasted about 25 min (Figure 1D). Sequence order was counterbalanced across participants.

We performed a sound check prior to scanning and then checked in through the microphone with the participants during the scan if they did not appear to be compliant with the task. The voice cue was provided because we wanted the participants to keep their eyes closed throughout the scan so they would focus their attention on the tactile cues and sensation of these cues. The 3 s voice cue of the task instructions were included as a regressor of non-interest in the model.

Notes were taken by an observer in the scanning console room, regarding the task performance of participants.

### Foot-Tapping Variables

Using *a priori* determined timestamps, each block consisted of three cycles of the rhythm (Figure 1) and was analyzed to identify peaks in the foot-tapper angle time-course, indicating discrete taps.

#### Amplitude Variability

Within each block of three cycles, the standard deviation of amplitude measured performance consistency. We produced a standardized value of amplitude, reflecting differences between participants' maximum ROM, i.e., its height relative to the adjacent local minima. We divided the participant timecourse by the second-largest range achieved within a single block (to avoid referencing to occasional amplitude spikes) and multiplied by 20 degrees. This calibration was performed independently for every functional run.

#### Timing Accuracy

This metric was defined on a 0–100 point scale. Observed foot-tap timing was compared against one ideal tap cycle (Figure 1C). The best cycle in the observed taps was identified by computing the linear regression, in a sliding window, of five consecutive observed taps against the ideal cycle. The cycle with the highest *R*^2^ for its regression (i.e., the best rate-invariant correspondence with the ideal cycle) was noted and removed. The process was repeated until all qualifying observed cycles had been identified. *R*^2^ values were summed and divided by 3, for three perfectly-executed cycles, to calculate timing accuracy. If participants completed a fourth cycle in a block, a denominator of four was used.

## Image Acquisition

Neuroimaging data were collected on a 3T Siemens Trio scanner using a 12-channel head coil for functional and anatomical runs. In functional runs, 114 T2^*^-weighted echoplanar image volumes measuring BOLD contrast were collected using parallel imaging with an iPAT acceleration factor of two. Scan sequence parameters were: 55 contiguous, 3 mm slices in the axial plane, interleaved slice acquisition, repetition time (TR) = 3,000 ms, echo time (TE) = 24 ms, flip angle = 90°, bandwidth = 2,632 Hz/pixel, field of view (FOV) = 230 mm, matrix = 76 × 76, voxel size = 3.0 × 3.0 × 3.0 mm. At the beginning of the run, the scanner acquired 3 TRs which were discarded automatically. An anatomical image was collected using a high resolution MPRAGE scan sequence with 176 contiguous slices in the sagittal plane, single-shot acquisition, TR = 2,300 ms, TE = 2.89 ms, flip angle = 8°, FOV = 256 mm, matrix = 256 × 256, bandwidth = 140 Hz/pixel, voxel size = 1.0 × 1.0 × 1.0 mm.

Care was taken to ensure that the whole of the cerebellum was covered by the bounding box of the fMRI data acquisition for all subjects. The MR imaging parameters of the EPI sequence employed during fMRI contained 55 axial slices with 3 mm slice thickness. Coverage afforded by these parameters were enough to obtain whole brain fMRI data from all subjects.

## Image Preprocessing

Image preprocessing and statistical analyses were conducted with AFNI (50) and FSL 5.0 (51) software. Slice-time correction and motion correction was performed on the functional volumes using the AFNI function 3dvolreg. The six resulting motion vectors were incorporated into baseline GLM analyses. Artifacts due to head motion, circulatory activity, and other noise sources were removed using FIX (52). The anatomical image was skull-stripped using optiBET (53), corrected for intensity bias introduced by magnetic field inhomogeneity, and then transformed to MNI space using the FNIRT procedure, producing a non-linear transformation based on local spatial properties. Functional data were aligned to the anatomical image and transformed using the non-linear transformation and smoothed using an isotropic, 3D, 6 mm full-width-half-maximum (FWHM) Gaussian kernel. Signal intensities in each volume were scaled with z-transformation excluding the first six volumes from calculation of the mean and standard deviation, avoiding pre-steady-state outliers. Analyses were performed on scaled data.

TRs with head movement exceeding 0.3 mm were excluded for NT participants, and 0.5 mm for the clinical population.

## Analysis

### Participant Characteristics

Descriptive statistics were calculated. Independent-samples *t*-tests compared groups on continuous measures. Chi-square tests compared categorical variables.

### Task Behavior: Foot-Tapping Metrics

Two trial-level statistics, amplitude variability, and timing accuracy, were calculated for active blocks. Pearson's *r* determined correlations between foot-tapping metrics and performance on mobility tests to ascertain ecological validity of the foot-tapping task. Participant means for each condition were calculated and submitted to a 2 condition (EG vs. IG foot-tapping) × 2 group (NT vs. PD) factorial ANOVA. Contrasts are reported for each dependent variable and the 2 × 2 interaction: EG vs. IG within each group, and NT vs. PD with each condition and Bonferroni corrected.

### Imaging Data

Individual-level regression was performed by fitting a general linear model to the scaled time series. Stimulus onsets were convolved with AFNI's “block” kernel, to model the hemodynamic responses for the EG and IG task conditions, and the rest periods. The auditory presentations of instructions were modeled using a canonical gamma variate kernel, modeling the hemodynamic responses to transient events. Six regressors obtained from motion correction during volume registration were included in the baseline model to remove residual signal change correlated with movement. Scanner drift was modeled by finding the best-fitting polynomial function correlated with the preprocessed timecourse data. A generalized least squares time series fit estimated temporal autocorrelation. The general linear model included three contrasts: EG vs. Rest, IG vs. Rest, and EG vs. IG. Beta coefficients and associated t-statistics from these contrasts from each participant's regression were entered into a mixed effects group-level analysis, accounting for individual estimates and their variability (54). Familywise error (FWE) corrected inferences were obtained through Monte Carlo (MC) simulation of the process of image generation, *estimated spatial correlation* of voxels, cluster detection thresholds and cluster identification (55) through the *ClustSim* program implemented in AFNI. This program assumes the underlying spatial correlation of the second-level analysis residuals is Gaussian (56, 57). The Gaussian spatial correlation employed by MC simulations was quantified by the average FWHM of first-level GLM analysis residuals across all participants (FWHMav = 8 mm). We acknowledge that the use of cluster size for statistical power has received some attention in related literature. A study by (58) reported that a cluster-based threshold of *p* < 0.001, when using a Gaussian kernel, may lead to false positives (58). In *consideration* of this report, we used an *updated version* of AFNI's 3dClustSim (Compile Date: 08/2016), using voxel padding to address edge effects associated with random field cluster generation. Recent studies have shown that the selected voxel cluster threshold using this technique avoid introduction of false positives as reported by (58) [see (55–57, 59)]. Voxels passing the cluster detection threshold, which was a family wise error-corrected cluster-level of *p* < 0.05, were considered to form a cluster if they shared a face.

For an ROI based analysis of the task × group interaction in the cerebellum, an adaptive Bonferroni method was used. For significance criterion α = 0.05 and *k* = 5 tests of interest (PD-IG vs. PD-EG, NT-IG vs. NT-EG, PD-IG vs. NT-IG, PD-EG vs. NT-EG, and the interaction), the smallest *p*-value was α/k = 0.01. The smallest remaining *p*-value was α/(k-1) = 0.0125, followed by α/(k-2) = 0.0133 and further until either a test failed to meet the criterion or all tests met the criterion.

## Results

### Foot-Tapping Task Performance

#### Ecological Validity of the Foot-Tapping Task With Measures of Gait and Balance

Foot-tapping performance measures were correlated with preferred and fast gait speed, and the DGI for EG and IG conditions (*r* = 0.31 to 0.49).

The foot tapping test was shown to have validity with measures of gait and balance in people with PD, as **Table 3** shows. Preferred and fast gait speed, the Dynamic Gait Index (DGI), and the Fullerton Advanced Balance (FAB) test were significantly correlated with timing and amplitude of the foot tapping task during EG and IG conditions. Timing during IG and EG conditions was significantly correlated with the Timed Up & Go (TUG) and the TUG-dual cognitive (TUG-c, counting backward by serial 3 s) measures; whereas only amplitude during IG was significantly correlated with TUG and TUG-c (**Table 3**).

#### Kinematics of Foot Tapping

ANOVAs revealed a significant task-by-group interaction in amplitude variability (*F* = 7.35, *p* = 0.009). Pairwise comparisons subjected to a Bonferroni correction (*p* = 0.0125) revealed that NT participants were less variable than PD participants in the IG condition [NT: M (SEM): 2.8 (0.22); PD: 3.6 (0.12); *p* < 0.001]. No significant differences in amplitude variability between groups in the EG condition [NT: 3.2(0.20); PD: 3.4 (0.14); *p* = 0.3] kept with the hypothesis that PD patients can perform EG tasks similarly to controls (10). The PD group was less accurate in timing for EG [NT: 57.1 (3.4)%; PD: 45.2 (3.4)%; *p* = 0.016] and IG tasks [NT:74.5(3.0)%; PD: 65.2(3.4)%; *p* = 0.044], but differences did not survive Bonferroni correction. Timing accuracy was greater for both groups during the IG task (*p* < 0.001).

#### Observed Task Performance of Participants

Field notes taken during the scans regarding the performance of participants revealed that overall participants performed the task as directed. Two individuals who reported weekly freezing of gait and exhibited some freezing of gait during the UPDRS-III exam performed the task with several errors; however, one participant with frequent freezing of gait and who exhibited FOG during straight walking performed the task with no errors. The level of freezing in the group is shown in Table 1.

### Imaging

#### Motor Network Activation Common to All Conditions

**Figure 2:**
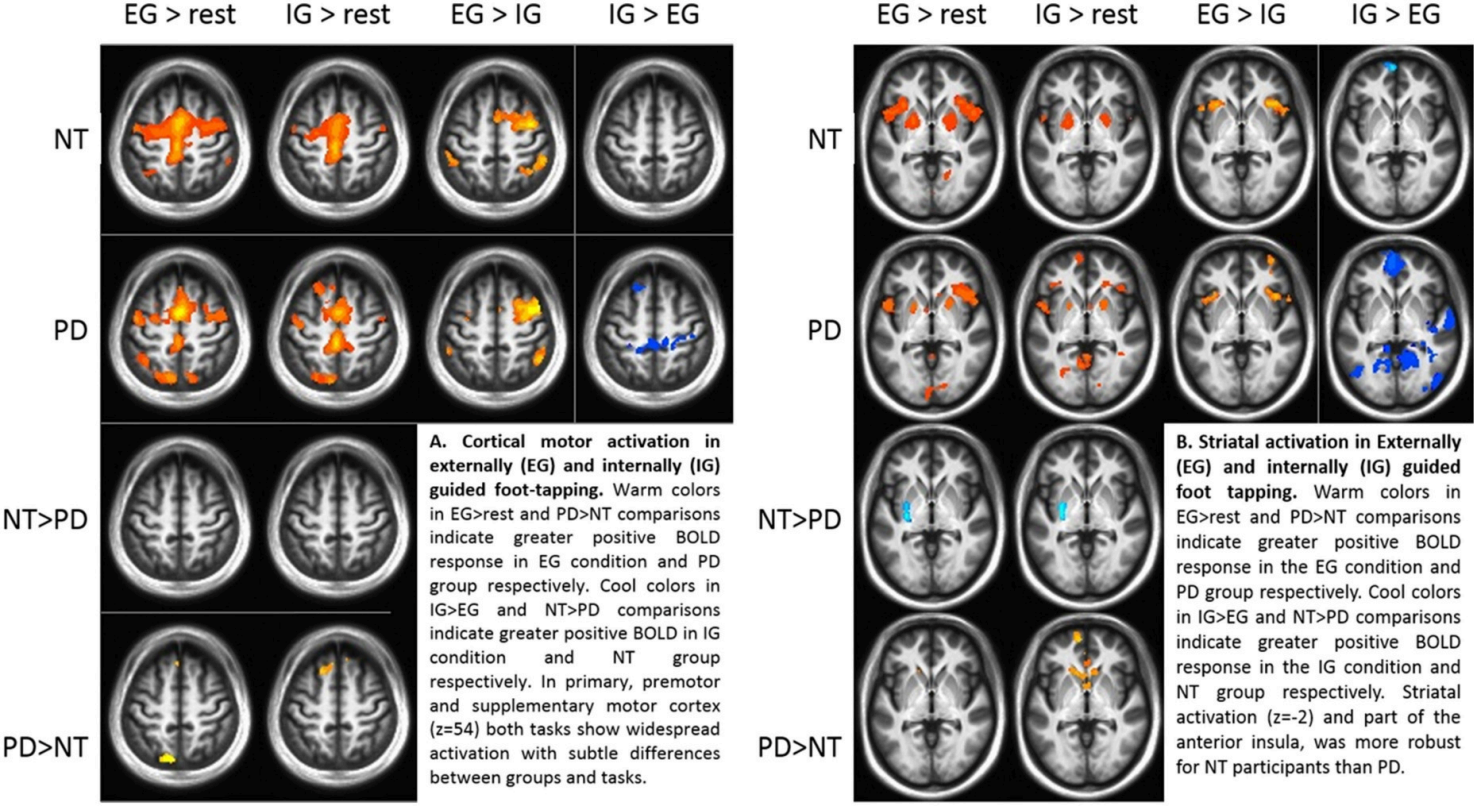
**(A)** Cortical motor activation during externally (EG) and internally (IG) guided foot-tapping. Warm colors in the EG>rest and PD>NT comparisons indicate greater positive BOLD response in EG condition and PD group respectively. Cool colors in IG>EG and NT>PD comparisons indicate greater positive BOLD in IG condition and NT group, respectively. In primary, premotor and supplementary motor cortex (*z* = 54) both tasks show widespread activation with subtle differences between groups and tasks. **(B)** Striatal activation for EG and IG guided foot tapping. Warm colors in the EG>rest and PD>NT comparisons indicate greater positive BOLD response in the EG condition and PD group, respectively. Cool colors in IG>EG and NT>PD comparisons indicate greater positive BOLD response in the IG condition and NT group, respectively. Activation in the striatum (*z* = −2) as well as part of the anterior insula, was more robust for NT participants than those with PD.

Comparing groups on EG and IG foot-tapping tasks against the resting baseline revealed robust activation throughout a motor network, including bilateral sensorimotor cortex, premotor cortex, the striatum (particularly putamen) (Figures 2A,B) and the cerebellum (Tables 2A,B). The left putamen was preferentially recruited by NT participants for EG (Table 2A) and IG tasks (Table 2B). **For the EG task**, clusters in the supplementary motor area (SMA), the caudate head, and the precuneus were preferentially activated for PD participants (Table 2A). **For the IG task**, activation for the PD group was more extensive in the caudate head and a left-lateralized set of frontal and parietal areas (Table 2B, Figures 2A,B).

**Table 2:** EG and IG foot-tapping vs. Rest and EG vs. IG foot-tapping in Neurotypical and PD Participants.

**Hem**.	**Region**	**BA/cereb**	**Vol. (mm^**3**^)**	**Peak *t***	**Peak voxel**
**(A) EXTERNALLY-GUIDED FOOT-TAPPING vs. REST**					
**NT**					
B	Motor, premotor, SMA putamen	4, 6	1,18,512	9.71	(−14,−4,52)
	IFG, DLPFC, insula	45, 44, 46, 13			
R	Cerebellum	multiple	16,632	6.51	(22,−34,−32)
R	Supramarginal g., pSTG	40	5,616	4.86	(54,−36,18)
R	Putamen, globus pallidus		4,384	5.69	(24,0,0)
R	Calcarine s.	17	4,376	5.02	(14,−78,2)
L	Supramarginal g.	40	3,520	4.27	(−38,−56,48)
L	pSTG	39	2,776	5.3	(−48,−36,22)
R	dlPFC	10	1,968	3.86	(36,42,24)
R	Cerebellum	VIIIa,b	1,648	4.8	(16,−60,−60)
L	Cerebellum	VI	1,520	3.77	(−28,−62,−30)
R	Cerebellum	VIIIa,b	1,336	3.67	(32,−70,−58)
L	Calcarine s.	17	1,312	3.77	(−18,−74,2)
L	Mid occipital g.	30	1,216	4.78	(−22,−64,34)
**PD**					
B	Motor, premotor, SMA putamen, caudate	4, 6	1,33,424	8.90	(4,2,58)
	IFG, DLPFC, insula	45, 44, 46, 13			
	dmPFC	8			
R	Cerebellum	multiple	14,656	6.19	(14,−38,−30)
R	Supramarginal g., pstg	40	8,312	5.47	(42,−42,40)
L	Supramarginal g.	40	6,728	5.23	(−38,−52,44)
B	Calcarine s.	17	6,400	4.9	(−6,−84,4)
L	Precuneus	7	4,232	7.3	(−12,−72,58)
L	Cerebellum	VI	1,320	6.71	(−38,−64,−28)
R	Precuneus	7	1,120	5.09	(16,−76,56)
**NT** **>** **PD**					
L	Putamen		3,152	−4.49	(−28,−4, 2)
**PD** **>** **NT**					
L	SMA	8	1,488	3.91	(−8, 34, 50)
L	Caudate head		1,048	4.35	(−12, 20, 4)
L	Precuneus	7	1,048	4.90	(−12,−76, 58)
**(B) INTERNALLY GUIDED FOOT-TAPPING vs. REST**					
**NT**					
B	Motor ctx., SMA premotor ctx. (L)	4,8,6	49440	8.44	(−4,−38,62)
B	Cerebellum	I_IV	9240	6.77	(16,−36,−28)
R	Premotor ctx.	6	4544	5.81	(56,−6,52)
L	pSTG	22	2760	4.19	(−48,−36,22)
L	Putamen, globus pallidus		2656	5.09	(−22,−2,−4)
R	Putamen		1808	4.56	(22,4,4)
L	Cerebellum	VI	1696	3.83	(−12,−72,−22)
R	Cerebellum	VIIIa,b	1568	4.39	(18,−42,−60)
R	Premotor ctx.	6	1248	4.64	(34,−12,46)
**PD**					
B	Motor ctx., SMA, dlPFC, dmPFC, caudate, putamen	4,6,8,9	108064	8.60	(−10,−46,74)
B	Cerebellum	I_IV	37528	7.35	(20,−44,−58)
L	Premotor ctx.	6	9872	5.96	(−60,0,18)
L	IPL, precuneus	40,39,7	7464	6.71	(−48,−76,36)
R	Premotor ctx.	6	6400	6.41	(62,6,16)
B	Calcarine s.	17	4032	4.46	(−2,−84,6)
L	cerebellum	VI,CrusI	2144	5.24	(−36,−64,−26)
R	Thalamus		1096	5.84	(8,−14,18)
**NT** **>** **PD**					
L	Putamen		1992	−3.96	(−30,−4,−2)
**PD** **>** **NT**					
L	dlPFC, dmPFC	9,8	10632	5.16	(−24,24,38)
B	Caudate, ACC	32	6296	5.01	(−14, 22, 2)
R	dlPFC	9	1784	4.57	(20, 42, 36)
L	Angular gyrus	39	1672	4.92	(−50,−68, 42)
L	vmPFC	10	1200	4.13	(−12, 62,−2)
**(C) EXTERNALLY-GUIDED vs. INTERNALLY GUIDED FOOT-TAPPING (EG vs. IG**)					
**NT (EG** **>** **IG)**					
R	Premotor cortex, dlPFC	6,9	32352	8.04	(32,0,52)
	- vlPFC (p. opercularis)	44			
	- Ant. insula	13			
B	- pre-SMA	6			
R	Inferior parietal lobule	40	7496	5.62	(52,−46, 52)
L	Ant. insula	13	3360	6.16	(−26,24,−8)
R	dlPFC	9	2224	4.74	(36, 42, 22)
L	Cerebellum	crusI,VIIb,VIIIa	2152	5.96	(−30,−44,−44)
R	Calcarine s.	17	1512	5.10	(14,−74, 6)
L	dlPFC	8	1176	4.88	(−30, 0, 70)
L	Inferior parietal lobule	40	1024	3.69	(−44,−46, 42)
**NT (IG** **>** **EG)**					
L	pMTG	39	2792	−6.23	(−54,−72, 26)
L	Lateral occiptal ctx.	19	1872	−4.57	(−46,−80,−16)
B	pre SMA - dmPFC, frontopolar ctx	10	1736	−7.54	(−4, 66, 26)
R	Lingual & fusiform g.	37	1344	−4.77	(30,−74,−14)
L	Retrosplenial ctx.	30	1288	−4.72	(−10,−60, 12)
L	Ventromedial PFC	11	1280	−5.1	(−4, 66,−2)
L	Parieto-occipital s.	23, 17	1112	−4.23	(4,−66,24)
B	Medial orbitofrontal ctx.	11	1080	−4.1	(4, 30,−24)
L	Mid. occipital g., IPL	19	1080	−4.58	(−32,−78, 38)
L	STS	21	952	−4.75	(62, 0,−14)
R	Mid. occipital g.	19	944	−4.49	(50,−80, 6)
L	Sup. occipital g.	19	808	−4.62	(−26,−96, 22)
**PD (EG** **>** **IG)**					
R	Premotor ctx., dlPFC	6,8	18816	7.37	(32,2,58)
R	Inferior parietal lobule	40, 39	10232	5.63	(50,−58, 54)
R	Ant. insula, vlPFC (p. opercularis.)	13,44	4952	6.38	(42,20,−8)
R	dlPFC, frontopolar ctx	9, 10	4664	5.13	(30, 48, 10)
L	Ant. insula	13	3080	6.43	(−40,16,−8)
R	Lateral OFC/vlPFC (p. orbitalis)	47	2848	4.55	(32, 66, 0)
L	Supramarginal g.	40	2592	5.24	(−54,−50, 46)
R	Pre-SMA, dorsal ACC	6, 32	2080	6.14	(6,14,42)
L	Premotor ctx.	6	1560	3.98	(−48,−2, 42)
L	dlPFC	8	848	5.83	(−30, 2, 70)
L	Cerebellum	crusII,VIIb	816	5.81	(−38,−46,−50)
**PD (IG** **>** **EG)**					
B	PCC, retrosplenial ctx.,	23,29,30,	90888	−9.12	(−48,−80,30)
	–	precuneus	31,		
	–	MTG, angular g.,	39		
	–	fusiform, parahippocampal g.	37,36		
	–	cerebellum	V,VI		
L	–	mid. occipital g.	19		
L	preSMA, vmPFC,	9,10,12,11	44360	−100	(−16,4,−42)
	–	lateral orbitofrontal ctx.	47		
B		–	medial orbitofrontal ctx.	11	
R	Sup. & mid. temporal G.	22, 21	6944	−7.07	(56,−12,−20)
L	Sup. & mid. temporal G.	22, 21	3424	−6.04	(−52,−6,−20)
L	Mid. & inf. occipital g.	19, 37	2016	−5.39	(−46,−68,−2)
R	Cerebellum	VIIIb	2000	−5.47	(12,−42,−54)
R	Somatosensory ctx./SII	3	1600	−4.85	(50,−12,22)
R	Posterior insula	13	1568	−5.24	(44,−18,−6)
R	Cerebellum	CrusI,II	1000	−5.12	(30,−86,−30)
R	Sup. occipital g.	19	952	−3.97	(22,−88,36)
R	Hippocampus (posterior)	808	−4.82	(36,−28,−14)	
**(D) TASK (EG vs. IG) X GROUP (NT vs. PD) INTERACTION**					
*(Parkinson's-EG + Neurotypical-IG) > (Parkinson's-IG + Neurotypical-EG)*					
NO CLUSTERS					
*(Parkinson's-IG + Neurotypical-EG) > (Parkinson's-EG + Neurotypical-IG)*					
L	Calcarine s., lingual g.	17, 18	3728	−4.25	(−28,−62, 0)
R	Calcarine s., lingual g.	17, 18	2936	−5.86	(10,−52, 0)
R	Cerebellum	crusII,VIIb,VIIIa	2120	−4.77	(20,−68,−46)
L	Posterior cingulate ctx.	23, 30	1424	−4.14	(−12,−46, 14)
R	Inferior temporal g.	21	944	−4.71	(42,−52,−4)

*All activation reported at FWE-corrected cluster-level p < 0.05. Hem., hemisphere (R, right, L, left, B, bilateral); BA/cereb, Brodmann's area/ Larsell lobules of cerebellum; p., posterior; ant., anterior; sup., superior; inf., inferior; mid., middle; s., sulcus; g., gyrus; ctx., cortex; dlPFC, dorsolateral prefrontal cortex; vlPFC, ventrolateral prefrontal cortex; SMA, supplementary motor area; pMTG, left posterior middle temporal gyrus; dmPFC, dorsomedial prefrontal cortex; g., gyrus; ACC, anterior cingulate cortex; PCC, posterior cingulate cortex; MTG, middle temporal gyrus; vmPFC, ventromedial prefrontal cortex; IFG, inferior frontal gyrus; pSTG, left posterior superior temporal gyrus*.

**Table 3:** Pearson's *r* correlations indicate the foottapping task has validity with balance and gait measures in participants with PD.

	**EG timing**	**IG timing**	**EG Amplitude**	**IG Amplitude**	**EG Amp SD**	**IG Amp SD**
Preferred gait speed	0.38^**^	0.34^*^	0.49^***^	0.48^***^	0.11	−0.04
Fast gait speed	0.31^*^	0.31^*^	0.36^**^	0.40^**^	0.04	−0.20
Dynamic Gait Index	0.39^**^	0.36^**^	0.44^***^	0.48^***^	0.07	−0.10
Fullerton Advanced Balance	0.31^*^	0.29^*^	0.31^*^	0.37^**^	0.06	−0.21
Timed Up & Go	−0.36^*^	−0.44^**^	−0.24	−0.37^**^	0.001	−0.07
Timed Up & Go–cognitive condition	−0.370^**^	−0.44^**^	−0.26	−0.39^**^	0.001	−0.08
UPDRS III	−0.19	−0.18	0.02	0.13	0.26	0.30^*^

*** *p < 0.001*,

** p < 0.01 and

* *p < 0.05*.

#### Differences Between EG and IG Conditions for Both Groups

Both groups' left cerebellar activation was stronger for the EG condition. Conversely, PD participants showed greater activity for IG than EG in the right (ipsilateral) cerebellum (Table 2C). Both groups' ipsilateral premotor activation was more robust in the EG condition (Figure 2A), as was activation bilaterally in the inferior frontal gyrus and intraparietal sulcus. The putamen was not preferentially activated during IG tapping for either group, but a set of occipital and medial cortical regions was activated for both groups. The extent of the activation clusters was greater for the PD group (Figure 2B: 2nd row, 4th column).

#### Task-by-Group Interaction in Right Cerebellum

**Figure 3:**
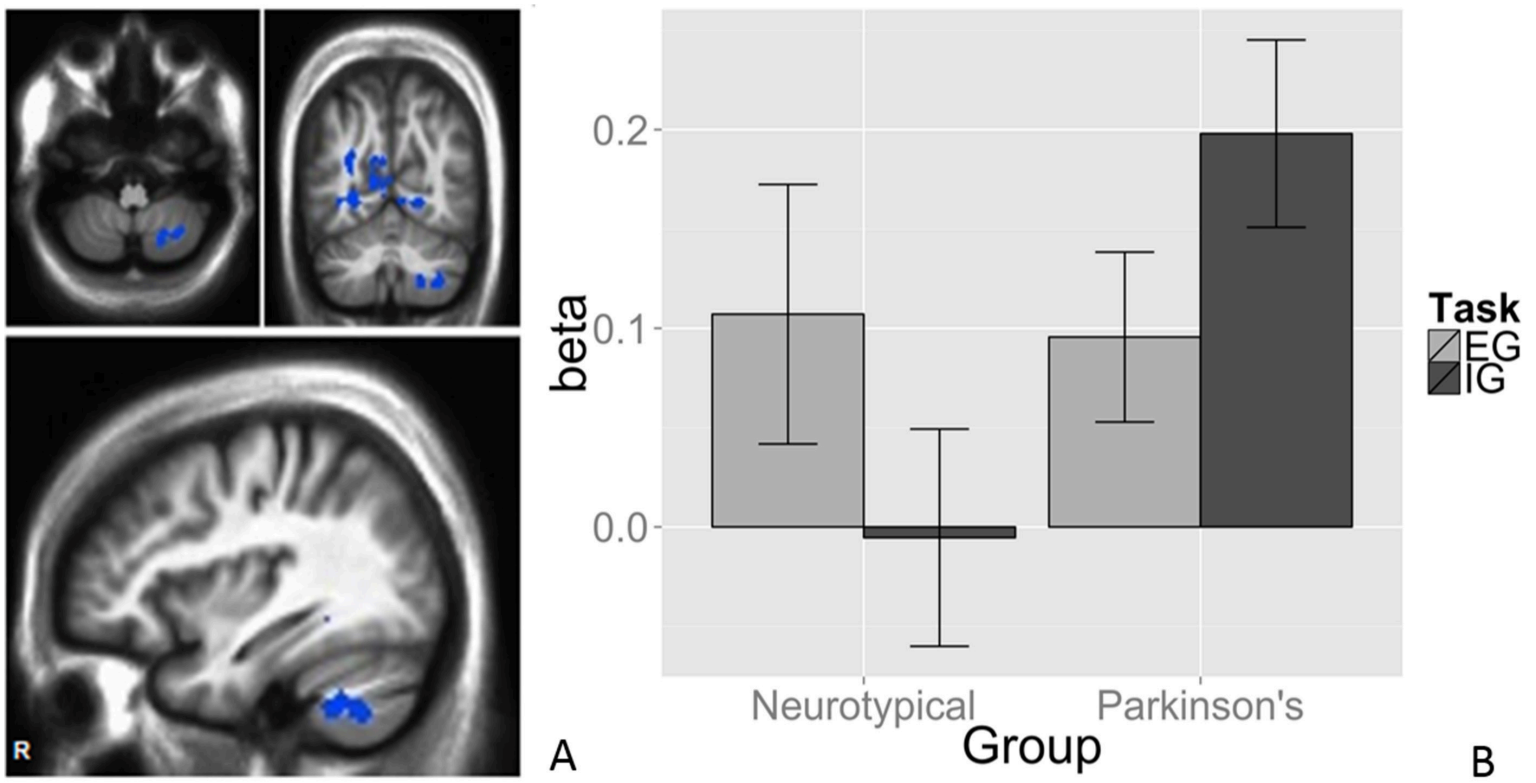
Task × group interaction in the cerebellum. Color contrasts illustrate group-by-task interaction in magnitude of BOLD response. Cool colors represent increased activation during the IG condition in the PD group, or during the EG condition in the NT group **(A)**. The significant interactions were driven by activation during the IG condition in the PD group **(B)**. Cerebellar differences between tasks were smaller in PD, suggesting an EG-like compensatory strategy for IG movement in participants with PD. The interaction was significant in Larsell Lobules, VIIb, VIIIa, and Crus II. MNI coordinates (36, −62, −50).

We observed a task-by-group interaction in the right cerebellum (Figures 3A,B). The 265-voxel cluster spanned portions of right crus II and lobules VIIb and VIIIa. Both groups activated the ipsilateral cerebellar cluster equivalently for the EG task (Figure 3B). During the IG task, the NT group was near baseline whereas the PD group recruited this cluster to a greater extent than during the EG task. Additional clusters significant for task-by-group interaction included loci in bilateral occipital and temporal cortices, and the left posterior cingulate (Table 2D). The cluster was subjected to pairwise contrasts of interest with respect to the hypotheses (Figure 3B). Observed betas for each condition for each subject were averaged across the cluster. Differences between each pair of conditions were calculated. An adaptive Bonferroni approach was used to correct for multiple comparisons as described in section 2.6.3. Three contrasts were statistically significant: EG-IG within PD (*p* = 0.0002, criterion α/k = 0.0125); EG-IG within NT (*p* = 0.0010, α/k = 0.0133); and NT-PD within IG (*p* = 0.0077, α/k = 0.0250), whereas NT-PD within EG was not significant (*p* = 0.8838, α/k = 0.0500). Activity during IG was significantly greater than EG for PD participants. Activity during EG was significantly greater than IG for NT participants. The IG condition showed significantly greater activity for PD participants. There were no group differences for the EG condition.

### Correlations Between Behavioral Foot Tapping Performance Metrics and Neural Activity

To assess the relationship between performance on the task and the observed neural effects, correlations were examined in four regions of interest, determined a priori and defined functionally. These correlations were performed for completeness in verifying or refuting the hypotheses. The left and right putamen were reliably activated in all four conditions vs. rest. The left cerebellum ROI was in a different location for each group, so the relevant EG > IG cluster was used for each group's analysis. Finally, the right cerebellum ROI was the one found significant in the task by group interaction.

For each ROI, correlation coefficients were calculated across subjects within each group (NT and PD), such that both performance metrics (timing accuracy and amplitude variability) were correlated against GLM contrast betas for each of the two tasks (EG and IG) vs. resting baseline (Table 4). None of these comparisons survived a Bonferroni correction.

**Table 4:** Correlations between foot tapping performance and neural effects.

	**L Putamen**	**R Putamen**	**L Cerebellum**	**R Cerebellum**
**NEUROTYPICAL EXTERNALLY GUIDED**				
Timing	−0.11 (0.653)	−0.21 (0.406)	0.08 (0.75)	−0.07 (0.791)
Amplitude variability	0.27 (0.273)	0.33 (0.181)	−0.29 (0.248)	−0.15 (0.556)
**INTERNALLY GUIDED**				
Timing	−0.01 (0.953)	−0.01 (0.956)	−0.05 (0.854)	−0.03 (0.917)
Amplitude variability	−0.02 (0.944)	−0.07 (0.79)	−0.35 (0.149)	−0.15 (0.544)
**PARKINSONS EXTERNALLY GUIDED**				
Timing	−0.12 (0.629)	−0.07 (0.757)	−0.06 (0.811)	0.12 (0.605)
Amplitude variability	−0.11 (0.649)	−0.05 (0.843)	**−0.6 (0.005)**	−0.22 (0.349)
**INTERNALLY GUIDED**				
Timing	**−0.51 (0.023)**	−0.33 (0.15)	−0.17 (0.468)	−0.28 (0.234)
Amplitude variability	−0.33 (0.153)	−0.27 (0.248)	−0.34 (0.144)	0.09 (0.721)

*Correlations and p-values (in parentheses) between neural areas and foot tapping performance metrics. Bolded values indicate significant (p < 0.05) findings*.

## Discussion

This study is the first to demonstrate a task by disease state interaction in the cerebellum, providing evidence for a possible compensatory mechanism by which older adults with PD may partially circumvent cortico-striato-thalamic circuitry impaired by dopaminergic depletion during lower limb movement. Using a task based on a complex rhythm provides ecological validity not possible with the more commonly used simple isometrical beat timing task and suggests a therapeutic avenue. Tasks using complex rhythms can potentially be used to evaluate the effects of therapies involving such rhythms, e.g., music-movement training and dance-based therapies. It was surprising that the EG task had worse kinematic performance than the IG task in terms of timing. This finding is unexpected given the abundance of literature that suggests that EG movement, including timing and amplitude is facilitated by cues. However, previous studies have used very simple external cues in terms of rhythm. This task is one of the first to use a tactile cue with a more complex rhythm than previous studies. Importantly, in addition to interesting cortical involvement during the tasks, our findings lend additional support to research showing the cerebellum serves in a compensatory role to assist the dysfunctional basal ganglia during IG movements in people with mild-moderate PD.

### How do Observed Activation Patterns for the EG and IG Tasks in the NT Group Fit Hypothesized Mechanisms?

#### EG

Structures activated more during EG conditions in NT adults (the dlPFC, the anterior insula, pars opercularis of the inferior frontal gyrus, the IPL and the contralateral cerebellum) are related to sensory guidance of movement, beat perception, and using salience for attention-demanding tasks. Given task demands, these functions are plausibly recruited by NT adults.

These activation areas are like areas noted during EG tasks by other studies of activation during foot movement, including Sauvage et al. (60), Schwingenschuh et al. (33), and Trinastic et al. (61), and Ciccarelli et al. (62). Suggesting the participants may have been imagining the experimenter as he or she cued their EG movements, this task activated the dlPFC and areas activated during motor imagery, rather than motor execution in other work (60). Other studies showed anterior insular activation in subjects hearing a musical piece after 30 min of actively learning a rhythmic melody (63), like the rhythmic task our participants learned before scanning.

#### IG

The structures comparatively activated more during IG conditions in NT adults [the middle temporal gyrus (MTG), the occipital gyri, the lingual and fusiform gyri, the retrosplenial cortex, the IPL and superior temporal sulcus, and the SMA] relate to mental imagery, planning complex behaviors, and extinction of conditioned responses. Participants needed to recall and perform a practiced task according to internal timing in IG tasks, meaning these functions are plausible. These activated areas are similar to areas noted during IG tasks by others (64, 65), and are common with areas recruited in motor imagery (66) (i.e., activations in the bilateral middle occipital gyrus and the lingual gyrus). Consistent with the present findings, prior research has found that movement paradigms where participants are free to choose their actions elicit activation in the preSMA, not the SMA (49, 67). Conversely EG movement paradigms have been shown to implicate the premotor cortex, a finding that was also replicated here (Table 2C).

We noted the cerebellum was activated more during EG in NT participants, but in the contralateral cerebellum (left side). However, within the NT group, the EG vs. IG contrasts did not reveal a task effect in the putamen despite prior findings suggesting a favored role in IG movements (68, 69). Our task was more rhythmically complex than the continuation task used elsewhere (22, 68), potentially contributing to this discrepancy. Coordination to a more complex external cue could also have led to poorer performance, as reflected in the poorer behavioral performance on the EG task as compared to the IG task.

### What Are the Implications of Differing PD and NT Group Activation Patterns for EG and IG Tasks?

#### EG

During EG tasks, both groups activated similar frontal, premotor, and cerebellar areas; additionally, the PD group activated bilateral PMC, the supramarginal gyrus, the dorsal ACC, contralateral cerebellum (crus II), and the orbitofrontal and frontopolar cortices. Previous reports showed PD patients off-medication activated the supramarginal gyrus, the ACC, motor cortex, the SMA and cerebellum. Individuals with PD engage more frontal areas and additional cerebellar activation during EG foot movement (33). A similar pattern was noted in PD participants (tested both on and off anti-parkinsonian medication) vs. controls in an EG visually cued force task. Off-medication PD participants recruited additional areas of the bilateral cerebellum and primary motor cortex not seen in on-medication PD participants and controls (70). Cerasa et al. (22) reported PD patients and controls engaged similar neural networks in EG and IG movement during visually-cued finger tapping, yet the PD group showed greater activity in sensory and associative cortices.

#### IG

In the IG task, both groups activated the retrosplenial cortex, the MTG, the fusiform, the middle and superior occipital gyrus, the vmPFC, the medial orbitofrontal cortex, and the pre-SMA; the PD group additionally activated the superior and MTG more extensively, the cerebellum (V, VI, VIIIb, Crus I, II), secondary somatosensory cortex (SII), the posterior insula, and the posterior hippocampus. These PD-unique regions in the IG task (apart from the cerebellum) are related to memory, sound processing (STG), and touch (posterior insula). For IG tasks, SII and cerebellar pathways are recruited (1, 10). During EG movements PD patients activate cerebellar circuitry similarly to controls, confirmed by NT group results. However, in PD, cerebellar and striatal circuits are activated during IG tasks (1). PD patients could have greater recruitment of the hippocampus and engage in more robust replay of auditory and somatosensory cues encountered during training.

Rhythmic EG and IG movements engage generally similar neural networks in PD and healthy controls (22). However, within IG and EG tasks, the putamen was activated more for NT participants (1) Loss of dopaminergic innervation to the basal ganglia hinders processing in cortico-striatal circuits (71). The PD group preferentially recruited SMA, caudate, and precuneus relative to NT participants in EG tasks, similar to previous findings (10), and dlPFC, SMA, angular gyrus, caudate, ACC and vmPFC in IG tasks. Cortical midline regions, e.g., medial prefrontal cortex, the precuneus, and the left angular gyrus, comprise the default mode network; their activation may imply PD participant difficulty attending to challenging IG tasks.

### How Does Observed Cerebellar Activity Vary Between Tasks and Groups?

#### Role of Cerebellum

The left cerebellum activation pattern was enhanced for EG tasks vs. IG tasks in both groups (Table 2), supporting the proposed preferential role of the cerebellum in EG motor tasks and consistent with recruitment of the CTC circuit for IG tasks in the PD group (1, 10, 72). Cerebellar recruitment is preferential for, but not exclusive to, EG movement in controls. The task-generality of cerebellar circuits may explain the disease-related plasticity described below.

#### Interaction in R Cerebellum

The task-by-group interaction was observed in the right (i.e., ipsilateral) cerebellum, the part of the cerebellum most directly relevant to right-sided foot-tapping. Both groups showed activation during the EG task relative to rest, as expected in a structure spared in early, mild to moderate PD and implicated in EG movement (Figure 3). The IG task did not recruit this region for the NT group, but did for the PD group. The direction of the relationship was reversed and the IG task recruited the right cerebellar cluster more than the EG task. Spraker et al. noted increased recruitment of cerebellar structures and pathways with PD progression (34), possibly becoming pathological over time (73).

The double dissociation between task and group suggests a compensatory role for the right cerebellum in PD. As the IG rhythmic motor task relies on the contribution of a STC mechanism that is compromised in PD, a parallel CTC circuit is co-opted. Prior work demonstrated a compensatory role of cerebellar motor circuits in PD (2, 10, 22, 72). Findings suggest the compensatory shift is specific to IG motor tasks, posing implications for PD patients frequently impaired in IG tasks (2, 15, 16).

### Strengths and Limitations

This study had several limitations that restrict the generalizability of the study.

One of the main limitations is that the task performed in the scanner included only movements of the right foot while lying supine. Therefore, conclusions regarding actual walking are very limited, even though the task outcomes are correlated with gait activities. Human movement performed during daily activities is rarely purely internally or externally guided. The IG and EG conditions we presented to our participants are only proxies for purely internally generated and externally generated movements. However, there was a key difference between the IG condition and the EG condition. We asked participants to keenly follow the timing of the tapper during EG but told them explicitly to disregard the tapping they felt during the IG condition and instead to perform the task as practiced prior to entering the scanner. The attention to cue for timing vs. non-attention to cue resulted in an IG vs. EG comparison that approximates internally vs. externally guided conditions. Both conditions likely induced error monitoring. Neither task was trivially easy, and both produced mistakes. To control for the sensory experience of tapping during the EG condition, during the IG condition, participants received a tap to their hand immediately after their own foot taps. As such, internally generated foot taps did produce feedback. However, this feedback was the same every time and therefore predictable and did not provide any indication regarding the quality of the tap in terms of the performance variables, timing and amplitude.

It must be acknowledged that the EG condition included an element of reaction time, because participants were asked to tap the foot immediately after sensing a tap on their hand. Further, it is likely that all movements have an aspect of IG movement, but in this case, participants were told to pay strong attention to the cue on their hand to elicit performance, which is similar to prior studies that provided auditory, or tactile stimuli with an inherent rhythm that stimulated movement in the individual with PD.

Although the pathways considered here have been relatively well-researched in the upper limb, they have not been much studied in the lower limb. For a condition like Parkinson's, which gravely affects the lower limb motor system in a plurality of patients, leading to falls and associated sequelae, it is vital to have enhanced understanding of lower limb motor control. This understanding can only be gained by conducting careful studies of neural pathways associated with lower limb behavior. Studying lower limb movement in the scanner is inherently challenging but the methods adopted in this study made evaluating a moderately complex rhythm in the scanner possible. Adapting this complex rhythm for foot movement in the scanner is therefore novel. MRI does not lend itself to ecologically realistic motor tasks, thus techniques like functional near-infrared spectroscopy (fNIRS) and electroencephalography (EEG) could measure neural activity in freely-moving participants in real time (74). However, these surface measures are not sensitive to putaminal or other subcortical structure activities. Performance of the research assistant conducting the response to IG, and prompt for EG, movements was not measured, possibly introducing a confound if their performance varied systematically between the tasks and/or participants. We did not measure the performance of the research assistant themselves, i.e., we do not know to what degree their tapping deviated from the recorded tapping which they heard and were to approximate. As such, we also do not know to what extent any performance inconsistencies in examiner tapping affected the behavioral outcome measures. While we did not record the examiner delivery, the task was controlled and practiced, and every researcher achieved a level of proficiency in matching their timing to the external auditory source, similarly to what is capable of drummers, musicians in an orchestra, precision dancing, marching bands, etc. Given our interest specifically in tactile cues as a form of external guidance, it was important to have a human perform the tapping, rather than an automated, vibrotactile machine. Recording the performance of the human would have increased the validity of the study.

The groups were not gender matched, although there was not a statistically significant difference in sex distribution (Chi square statistic = 2.18, *p* = 0.14). However, the difference in numbers of males vs. females in each group could have affected the findings in unknown ways. That said, we are reasonably confident that the sex distribution in the current study represents the population well-given that the prevalence of males to females in the PD population is 60/40 (75) and our present sample is ~55%/45%. The study would have been better controlled if truly matched for gender, and certainly some work shows differences in neural control (e.g., visuospatial processing) based on sex or aging (76), but we do not see any reason that women and men would have performed the foot tapping task differently from one another in any systematic way, given that the amount of foot movement that is required is no more than that needed to drive or to use a piano pedal. Among classical musicians who play the piano, women and men are now considered to be equally able to take on all the most difficult repertory. Therefore, we believe there is little reason to suppose that the performance of this particular task would have differed for any reasons based on sex.

The sample size ultimately limits our ability to make definitive conclusions.

## Conclusion

As we hypothesized, similarly to finger-tapping tasks (1, 10, 35), this study shows IG and EG foot movement tasks are mediated by neural mechanisms involving the striatum and cerebellum, respectively, in healthy individuals without PD. However, PD may influence circuits involving the striatum or cerebellum on a context- and/or task-specific basis. Additional regions, e.g., the ipsilateral cerebellum, were recruited by PD participants to perform the IG tasks. The internally guided and externally guided conditions of the task investigated here provides insight into the breadth of neural structures involved in complex, rhythmic movement in individuals with and without Parkinson's disease.

## Ethics Statement

This study was carried out in accordance with the recommendations of the Emory University IRB and the VA R&D Review committees with written informed consent from all subjects. All subjects gave written informed consent in accordance with the Declaration of Helsinki. The protocol was approved by the Emory University IRB and the VA R&D Review committees.

## Author Contributions

The research project concept was defined by MH, KS, BC, DC, SW, JD, and ME. MH, KM, VK, and KG organized the project. The research project was executed by MH, JD, KM, AB, AH, and ME. MH, JD, VK, KM, and LK designed the statistical analysis plan. The statistical analysis plan was executed by JD and MH and received critiques from KS, BC, VK, SW, DC, KG, and ME. JD and MH wrote the first draft of the manuscript. KS, BC, VK, SW, DC, KG, AH, and ME reviewed and critiqued the manuscript.

### Conflict of Interest Statement

The authors declare that the research was conducted in the absence of any commercial or financial relationships that could be construed as a potential conflict of interest.
